# Supplementary material for: Elevated CLOCK and BMAL1 Contribute to the Impairment of Aerobic Glycolysis from Astrocytes in Alzheimer’s Disease
Source: Int J Mol Sci. 2020 Oct 23;21(21):7862. doi: 10.3390/ijms21217862 (PMC7660350; doi:10.3390/ijms21217862)
Supplement: Supplementary file 1 [file ijms-21-07862-s001.pdf]

Supplemental information

Elevated CLOCK and BMAL1 contributes to the impairment of aerobic glycolysis from astrocytes in Alzheimer's diseases.

Ik Dong Yoo [1], Min Woo Park [2], Hyeon Woo Cha [2], Sunmi Yoon [3], Napissara Boonpraman [3], Sun Shin Yi [3]*, and Jong-Seok Moon [2]*

Supplemental Table S1. The characteristics of patients with AD and non-AD donor (normal).

	**Normal**<br><br>**(n= 3)**	**AD**<br><br>**(n= 3)**
**Age, years**	65 ± 16	72 ± 11
**Sex, male/female**	2 / 1	0 / 3
**Brain weight (g)**	1300 – 1400	848 – 928

Figure S1

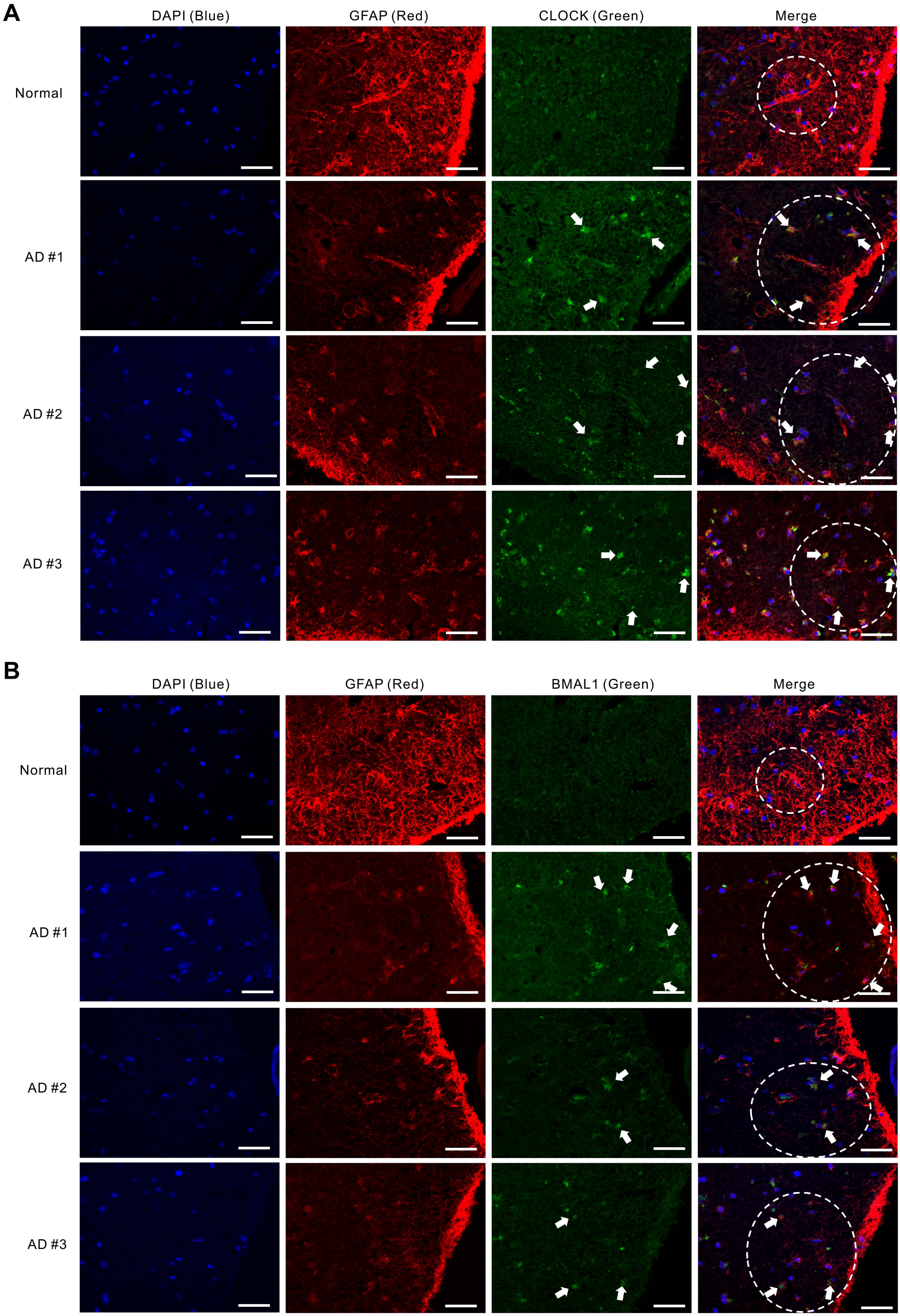
A
DAPI (Blue)
GFAP (Red)
CLOCK (Green)
Merge
Normal
AD #1
AD #2
AD #3
B
DAPI (Blue)
GFAP (Red)
BMAL1 (Green)
Merge
Normal
AD #1
AD #2
AD #3

Supplemental Figure S1. The levels of CLOCK and BMAL1 are elevated in impaired astrocytes in cerebral cortex region from patients with Alzheimer's diseases. (A) Representative immunofluorescence images of CLOCK expression in cerebral cortex region from patients with AD (AD) or non-AD (normal) showing CLOCK (green) in astrocytes expressing the astrocytes marker GFAP (red) around vessels. DAPI-stained nuclei are shown in blue. Scale bars, 20 μM. (B) Representative immunofluorescence images of BMAL1 expression in cerebral cortex region from patients with AD (AD) or non-AD (normal) showing BMAL1 (green) in astrocytes expressing the astrocytes marker GFAP (red) around vessels. DAPI-stained nuclei are shown in blue. Scale bars, 20 μM.

Figure S2

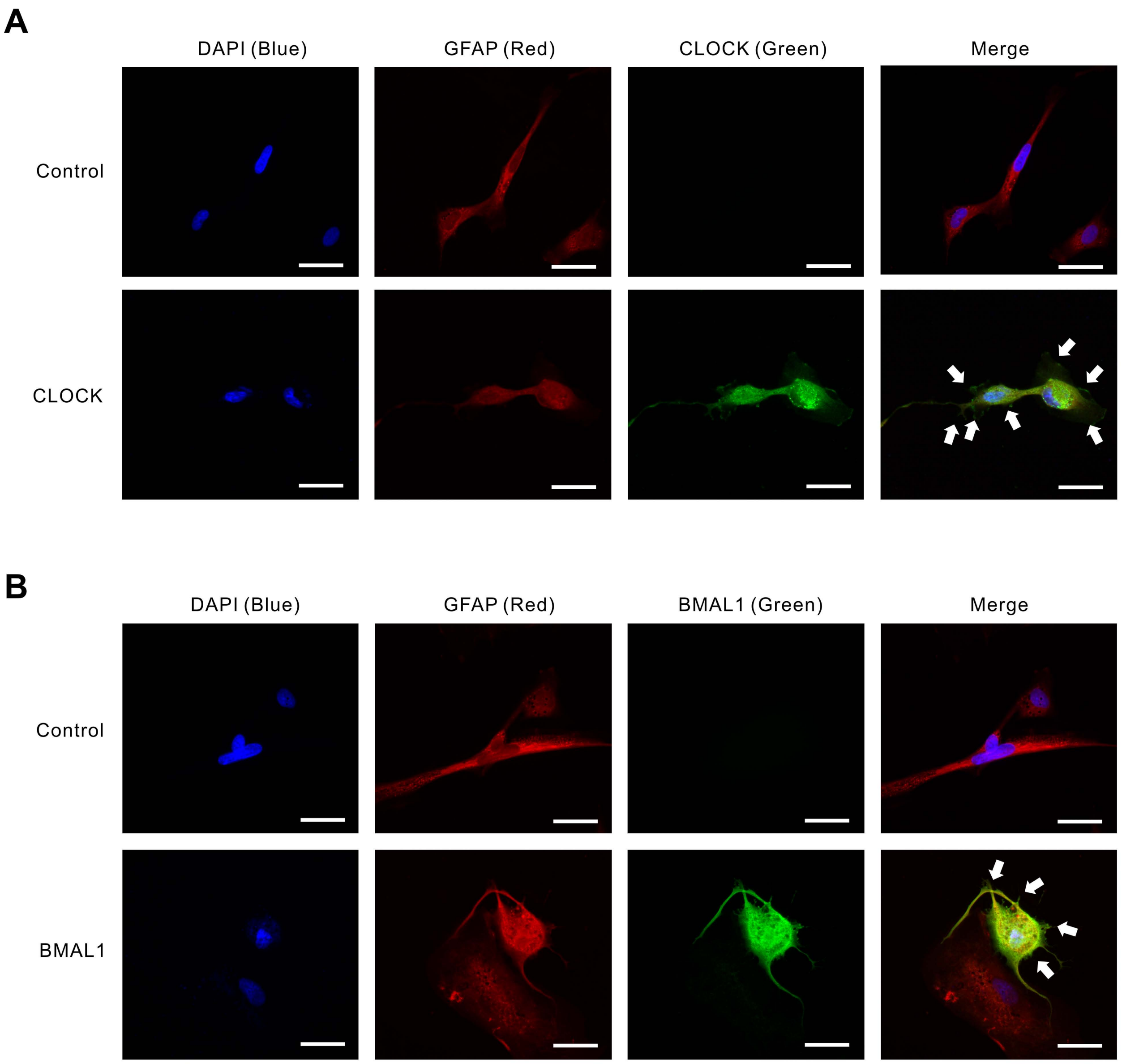

Supplemental Figure S2. The elevation of CLOCK and BMAL1 contributes to the functional impairment by reduction of GFAP-positive filaments in human astrocytes. (A) Representative immunofluorescence images of CLOCK expression in control and CLOCK overexpressing (CLOCK) human astrocytes showing CLOCK (green) and GFAP (red). DAPI-stained nuclei are shown in blue. Scale bars, 20 μM. (B) Representative immunofluorescence images of BMAL1 expression in control and BMAL1 overexpressing (BMAL1) human astrocytes showing BMAL1 (green) and GFAP (red). DAPI-stained nuclei are shown in blue. Scale bars, 20 μM.
